# Vitamin D and the Athlete: Risks, Recommendations, and Benefits 

**DOI:** 10.3390/nu5061856

**Published:** 2013-05-28

**Authors:** Dana Ogan, Kelly Pritchett

**Affiliations:** Department of Nutrition, Exercise and Health Science, Central Washington University, 400 E. University Way, Ellensburg, WA 98926, USA; E-Mail: kkerr@cwu.edu

**Keywords:** vitamin D, athletic performance, 25(OH)D, supplementation, insufficiency, athlete

## Abstract

Vitamin D is well known for its role in calcium regulation and bone health, but emerging literature tells of vitamin D’s central role in other vital body processes, such as: signaling gene response, protein synthesis, hormone synthesis, immune response, plus, cell turnover and regeneration. The discovery of the vitamin D receptor within the muscle suggested a significant role for vitamin D in muscle tissue function. This discovery led researchers to question the impact that vitamin D deficiency could have on athletic performance and injury. With over 77% of the general population considered vitamin D insufficient, it’s likely that many athletes fall into the same category. Research has suggested vitamin D to have a significant effect on muscle weakness, pain, balance, and fractures in the aging population; still, the athletic population is yet to be fully examined. There are few studies to date that have examined the relationship between vitamin D status and performance, therefore, this review will focus on the bodily roles of vitamin D, recommended 25(OH)D levels, vitamin D intake guidelines and risk factors for vitamin D insufficiency in athletes. In addition, the preliminary findings regarding vitamin D’s impact on athletic performance will be examined.

## 1. Introduction

As research has progressed, the importance and versatility of vitamin D in the body has become quite evident, therefore the prevalence of vitamin D insufficiency has been heavily examined in recent years. Research suggests vitamin D’s active role in immune function, protein synthesis, muscle function, inflammatory response, cellular growth and regulation of skeletal muscle [1,2,3,4]. In addition, a common symptom of clinical vitamin D deficiency is muscle weakness. Due to the many essential roles of vitamin D within the body, it has been suggested that physical performance may be influenced by serum vitamin D status, especially in those who are clinically deficient.

Vitamin D insufficiencies are estimated to affect over one billion people worldwide [5]. The Third National Health and Nutrition Examination Survey (NHANES III) data showed a significant increase in vitamin D insufficiency in the USA over the last 30 years, with over 77% of Americans considered vitamin D insufficient [6]. The alarming rates of insufficiency and the vast metabolic properties of vitamin D have led researchers to examine the influence of vitamin D, not only on disease prevention, but also on physical performance and injury. Vitamin D has been identified in most tissues within the body, including skeletal muscle, which has led to further examination of vitamin D’s influence on athletes and physical performance.

Because athletes and sports medicine physicians are primarily concerned with performance, the risk of vitamin D insufficiency among athletes has received growing interest and is under current examination by many researchers. In the last decade, researchers have examined 25(OH)D levels among various groups of athletes, ranging from gymnasts to runners to jockeys. Some findings have suggested that vitamin D levels in athletes are comparable to those of the general population; however, results depended largely on geographical location and type of sport (indoor *vs.* outdoor). It is apparent that the athlete is at an equal risk for vitamin D insufficiency, therefore the potential impact of vitamin D status on performance is now under examination. There are few studies to date that have examined the relationship between vitamin D status and performance, therefore, this review will focus on the bodily roles of vitamin D, recommended serum 25(OH)D level, vitamin D intake guidelines and risk factors for vitamin D insufficiency in athletes. In addition, the preliminary findings regarding vitamin D’s impact on athletic performance will be examined. 

## 2. Physiology & Bone Health

Vitamin D functions in two distinct ways within the body, through endocrine and autocrine mechanisms. The first, and most well-known, mechanism is the endocrine function, which enhances intestinal calcium absorption and osteoclast activity [1]. Vitamin D is essential for bone growth, density and remodeling, and without adequate amounts, bone loss or injury will occur [7]. When vitamin D is low, parathyroid hormone (PTH) increases to activate bone resorption in order to satisfy the body’s demand for calcium [8]. Low vitamin D increases bone turnover, which increases the risk for a bone injury, like a stress fracture. 

A study examining male Finnish military recruits found vitamin D status to be a significant determinant of maximal peak bone mass and also discovered that 25(OH)D levels below 30 ng/mL significantly increased the risk of stress fractures in this subject group [9]. In a large (*n* = 3700) vitamin D supplementation trial using female navy recruits, subjects receiving 800 IU/day of vitamin D for eight weeks, had a 20% lower incidence in stress fractures than the placebo group [8]. These studies in active populations, such as military recruits, display the critical role that vitamin D plays in optimal bone health. These findings also suggest that sufficient vitamin D status may prevent injuries, such as stress fractures. Stress fractures are quite common among athletes; most commonly seen among track and field sports, in up to 10%–31% of athletes [8]. Stress fractures can significantly influence performance due to debilitating pain and even cause permanent disability [8]. 

Vitamin D’s other pathway is the autocrine pathway. It is less recognized, but many essential metabolic processes take place in this pathway. On a daily basis, over 80% of the vitamin D within the body is utilized through the autocrine pathway [10]. The autocrine pathway is involved in essential body processes like signaling gene response/expression, synthesizing proteins, hormone synthesis, immune/inflammatory response, plus, cell turnover and synthesis [10]. “Without vitamin D, the ability of the cell to respond adequately to pathologic and physiologic signals is impaired” [10].

## 3. Vitamin D and Muscle Tissue

The autocrine pathway appears to be of utmost importance and has recently received a great deal of attention in regards to vitamin D’s influence on skeletal muscle function [11]. Vitamin D receptor (VDR) sites have been identified in virtually every tissue within the body [12]. VDR regulates expression in hundreds of genes that perform essential bodily functions. The discovery of VDR within the muscle suggested a significant role for vitamin D in muscle tissue and has since been identified as a regulator of skeletal muscle [3,11,13,14,15,16]. There are two proposed mechanisms by which vitamin D status may influence muscular strength. One possible explanation involves the direct role of 1,25-dihydroxyvitamin D [1,25(OH)2D] on VDRs within the muscle cells [11,17,18]. A second explanation suggests that vitamin D modifies the transportation of calcium in the sarcoplasmic reticulum by increasing the efficiency or number of calcium binding sites involved in muscle contraction. This indirect mechanism however, has only been examined in rat models [11]. On the contrary, one study disputes the evidence for the presence of VDRs within the skeletal muscle cells and suggests that the immunocytochemical staining to detect VDR may be responsible for the false positives results in previous studies [18,19].

Furthermore, it has been suggested that vitamin D supplementation in individuals with low vitamin D status may improve muscle strength. This is believed to be due to an increase in the size and amount of type II (fast twitch) muscle fibers associated with vitamin D supplementation [11,20]. It should be noted that type II fibers are predominant in power and anaerobic activities, and are recruited first to prevent falls, associated with muscle strength in the aging population [11]. 

Various researchers have found vitamin D to have a significant effect on muscle weakness, pain, balance and fractures in aging individuals [3,4]. It is difficult, however to compare the results given the variety of outcome measures and differences in populations used in the studies [14]. Several observational studies have suggested that vitamin D status influences muscular strength and function in the elderly [11,21]. Contrary to these findings, Chan *et al.*, (2012) found no association between baseline vitamin D status and changes in performance measures over a four year period [14,22]. Replacing vitamin D stores in the elderly population may be protective against fall risk and declining physical function [11,14]. 

Few studies to date have examined this relationship in the adolescent population. Foo *et al.* (2009) examined the relationship between 25(OH)D status and bone mass, bone turnover, and muscle strength in Chinese adolescent females (*n* = 301) and found that poor vitamin D status (<20 ng/mL) was associated with reduced forearm strength, (using a handgrip dynanmometer) when compared to individuals with adequate vitamin D levels (>20 ng/mL) [17]. Ward *et al.* (2004) suggested that 25(OH)D levels were positively associated with muscle power, and jump height in postmenarchal females (*n* = 91), however physical activity levels were not taken into consideration [11,23].

These findings in regard to muscle tissue and function suggest that vitamin D status may have a significant effect on muscle performance and injury prevention, therefore possibly influencing athletic performance. However, further research is warranted to determine the magnitude of effect of vitamin D on muscle strength and performance.

## 4. Vitamin D Recommendations (Intake and Desirable Levels)

Although the sun is the most plentiful source of vitamin D, there are also some dietary sources. Some common foods contain significant levels of vitamin D, naturally, including salmon, fatty fish, egg yolks, plus, fortified products also exist, such as, milk, cereal and orange juice [24]. While these dietary sources may appear significant, the process of absorbing dietary vitamin D is only about 50% efficient; therefore, much of the nutrient value is lost in digestion [25]. The lack of dietary vitamin D is yet another factor that increases the risk of vitamin D insufficiency. Most experts agree that a higher intake of vitamin D, through dietary sources, ultraviolet B (UVB) exposure, and supplementation, is necessary to obtain optimal serum vitamin D levels [10,26,27,28].

In November of 2010, the Institute of Medicine (IOM) released new recommendations for dietary intake of vitamin D, 400–600 IU/day for children & adults (0–70 years), 800 IU/day for older adults (>70 years) [29]. These values are only slightly higher than past recommendations [29]. Many experts argue that while IOM intake recommendations may adequately prevent clinical vitamin D deficiency, they are significantly lower than the level necessary to achieve optimal vitamin D status [5,6,10,26]. The Recommended Dietary Allowance (RDA) for Vitamin D, according to the National Institute of Medicine (IOM) [29] is compared to the Endocrine Society’s [30] recommended intake in Table 1. Many believe that the RDA is grossly underestimated [5,6,10,26], including the Endocrine Society, who released vitamin intake guidelines that are significantly higher [30]. The Endocrine Society recommends 400–1000 IU/day for infants, 600–1000 IU/day in children (1–18 years) and 1500–2000 IU/day in adults, in addition to sensible sun exposure [30]. 

Another area of debate among vitamin D researchers is the terminology and reference values used to define optimal vitamin D status, deficiency, and insufficiency. Optimal serum 25(OH)D concentrations have yet to be defined; however, most researchers have similar reference values [31]. Vitamin D deficiency is often defined as <20 ng/mL (50 nmol/L), and insufficiency defined as 20–32 ng/mL (50–80 nmol/L) and optimal levels are >40 ng/mL (100 nmol/L) [5,10,12,32]. The term insufficiency “appears to be the currently favored term for the range of marginal deficiency and is the theoretical serum concentration that is not high enough to protect against certain chronic diseases” [32].

**Table 1:** Recommended vitamin D intake levels of the Institute of Medicine *vs.* Endocrine Society [29,30].

Age	Recommended Intake (IU/day)	Upper Limit (IU/day)
**National Institute of Medicine**		
Children (0–18 years)	400–600	2500 (1–3 years) 3000 (4–8 years) 4000 (13–18 years)
Adults (19–70 years)	600	4000
Older Adults (>70 years)	800	4000
Pregnancy/Lactation	600	4000
**The Endocrine Society**		
Children (0–18 years)	400–1000	2000–4000
Adults (19–70 years)	1500–2000	10,000
Older Adults (>70 years)	1500–2000	10,000
Pregnancy/Lactation	600–1000 (14–18 years) 1500–2000 (19–50 years)	10,000

Optimal levels of serum 25(OH)D are no exception to the controversy. When serum levels reach >32 ng/mL, parathyroid hormone (PTH) levels become stable and reduce the risk of secondary hypoparathyroidism, which is commonly associated with low vitamin D status. In addition, intestinal calcium absorption is enhanced, reducing the risk of secondary bone disease [5,28]. These basic vitamin D functions are efficiently demonstrated at 25(OH)D levels >32 ng/mL; however, superior benefits are observed at even greater levels. For example, only at 25(OH)D levels >40 ng/mL, does vitamin D begin to be stored in the muscle and fat for future use [20,28]. Therefore, at levels <40 ng/mL, the body relies on a daily replenishment of vitamin D to directly satisfy its daily requirements, which is not likely to be present in the common diet. At levels <40 ng/mL, there appears to be just enough circulating 25(OH)D available for all of the immediate metabolic needs; however, stored vitamin D is not likely available for the advanced processes involved in the critical autocrine pathways [20]. 

It is estimated that the body requires 3000–5000 IU of vitamin D per day to meet the needs of “essentially every tissue and cell in the body” [12]. The IOM recommends 600 IU of vitamin D for most adults (18–70 years of age) to prevent clinical vitamin D deficiency, defined as 25(OH)D ≤ 20 ng/mL [29]. In contrast, most expert’s recommendations are much higher than 600 IU per day, because their recommendations are designed to help reach optimal 25(OH)D levels of at least 40 ng/mL. Intake levels recommended by most experts not only allow support for daily metabolic requirements, but also allow for vitamin D storage and increased availability, which appears to reduce the risk of many diseases and possibly enhance performance. The recommended daily vitamin D intake, according to most experts, is at least 1000 IU per day to maintain optimal 25(OH)D status; however, more is required if levels begin suboptimal [5,10,28]. With over 77% of Americans considered insufficient in vitamin D, it is apparent that the current recommendations are suboptimal [5,6,10,26].

Intake recommendations increase with age, pregnancy, and lactation. In addition, experts recommend much higher initial dosages if 25(OH)D levels begin deficient, ranging from 2000 to 200,000 IU, until optimal 25(OH)D levels are met, then 1000–2000 IU/day for maintenance [5,28,32]. A commonly prescribed treatment to quickly correct vitamin D deficiency is a weekly dose of 50,000 IU of vitamin D for eight weeks [12].

The tolerable upper limit for vitamin D has been set by the IOM at 4000 IU for adults, compared to 10,000 IU/day by the Endocrine Society [29,30] (Table 1). Leading experts have claimed that a daily intake of 10,000 IU would take months, or even years to manifest symptoms of toxicity [28]. A recent publication found no cases of toxicity with daily intakes of 30,000 IU per day for an extended period of time [10]. Regardless of the current dietary intake value, the amount of vitamin D produced from 15 min of unprotected sun exposure is 10,000 to 20,000 IU, in a light-skinned individual, making most experts believe toxicity to be a rare and unlikely event [10,12]. During the months that UVB rays are available from the sun, five to 15 min of unprotected sun exposure between the hours of 10 a.m. and 3 p.m. appear to provide adequate amounts of vitamin D [12]. 

There have never been any reported cases of vitamin D toxicity from over exposure to the sun; however, symptoms of intoxication, such as hypercalcemia, have been observed when 25(OH)D levels are greater than 150 ng/mL [12]. Serum 25(OH)D levels in individuals living close to the equator and working outdoors are often around 50 ng/mL, supporting the theory that vitamin D toxicity from the sun is extremely unlikely, and suggesting that any toxicities would result only from over supplementation [28]. Regardless, many experts agree than 1000 IU/day in the absence of proper sun exposure can maintain 25(OH)D levels of at least 32 ng/mL [12]. 

## 5. Vitamin D Status of Athletes

The distance from the equator, season, and time of day dictate whether vitamin D is available from the sun. Production of vitamin D from the sun is also dictated by cloud cover, pollution, sunblock, skin pigment and age. During the summer months, UVB radiation from the sun can be absorbed in adequate amounts to synthesize vitamin D [5]. However, during winter months, the angle of the sun prevents UVB radiation from reaching latitudes greater than 35–37 degrees, therefore, vitamin D cannot be synthesized from in these areas [5,20]. 

Research has suggested that low levels of vitamin D are widespread in populations living south of the 35th parallel [26]. Even if one spends ample time in the sun, sunscreen with a sun protection factor (SPF) of 15 results in a 99% decrease in vitamin D absorption [5]. Individuals who spend ample time outdoors may still need vitamin D supplementation to maintain adequate levels during the winter [33,34]. Many outdoor athletes avoid peak sunlight hours, opting to practice early in the morning or late at night, which greatly reduces UVB exposure, putting them at considerable risk of vitamin D insufficiency. Various studies have found many athletes to be at high risk for vitamin D insufficiencies. Table 2 displays prevalence of vitamin D insufficiencies among diverse athletic groups.

Hamilton *et al.* (2009) revealed that 90% of Middle Eastern sportsmen were vitamin D deficient between April and October [33]. Although these sportsmen were training at favorable latitudes, Qatar (25.4°N), they averaged less than 30 min of sun exposure per day. Another study conducted at favorable latitude (Israel 31.8°N), suggested that 73% of athletes were vitamin D insufficient [35]. The majority (83%) of female, Australian indoor athletes were also found to be vitamin D insufficient [36]. In contrast, a study conducted at less favorable latitude (Laramie, WY 41.3°N), revealed vitamin D insufficiency in 63% of indoor/outdoor athletes during winter, compared to the fall (12%) and spring (20%) in indoor and outdoor athletes [37]. Finally, a study conducted even further from the equator (Ellensburg, WA 46.9°N), using exclusively outdoor athletes, found 25%–30% with vitamin D insufficiency from fall to winter [38]. Storlie *et al.* suggested that 1000 IU/day of vitamin D was not enough to prevent seasonal decline of vitamin D status in this cohort [38]. Although the results are variable, geographical location (latitude) and gender do not appear to be the major risk factors for vitamin D insufficiency in athletes. Lack of sun exposure appears to be the main risk factor, putting indoor athletes and those who avoid peak daylight hours, regardless of latitudinal location, at the greatest risk for vitamin D insufficiency [2,9,33,35,36,37,38].

**Table 2:** Prevalence of Vitamin D deficiency (<20 ng/mL) and insufficiency (<32 ng/mL) in various athletic populations.

Type of Athlete	Indoor/Outdoor	Gender	Vitamin D Status	Reference
Finnish military recruits	Combination	Male	39% deficient	Valimaki *et al.* [8]
UK professional athletes (jockeys, rugby, soccer)	Combination	Male	62% deficient	Close *et al.* [39]
UK athletes (football, rugby)	Combination	Male	57% deficient	Close *et al.* [40]
Middle Eastern sportsman	Combination	Male	32% insufficient 58% deficient	Hamilton *et al.* [33]
Australian gymnasts	Indoor	Female	33% insufficient	Lovell [36]
Israeli athletes & dancers	Indoor	Male & Female	73% insufficient	Constantini *et al.* [35]
USA indoor/outdoor athletes	Combination	Male & Female	12% insufficient	Halliday *et al.* [37]
USA endurance athletes (runners)	Outdoor	Male & Female	42% insufficient 11% deficient	Willis *et al.* [2]
USA outdoor athletes (rugby, football, track, cross country)	Outdoor	Male	25% insufficient	Storlie *et al.* [38]

## 6. Vitamin D and Athletic Performance

Original research concerning vitamin D and athletic performance dates back to the early twentieth century, but current performance trials are quite limited and inconclusive. Russian and German researchers were the first to report the convincing effects of ultraviolet light irradiation for improving athletic performance and decreasing chronic sports related pain [20]. These early European researchers suggested significant improvements in time trials, cardiovascular fitness, and strength with treatment of UVB irradiation prior to performance [20]. German Olympic officials considered these effects significant enough for UVB radiation (vitamin D) to be considered an ergogenic aid. In support of this concept, many athletes claim to peak in physical fitness during the time of year that vitamin D (UVB) levels are at their highest, summer and fall [20]. 

Unfortunately, there are limited experimental studies available and even fewer that demonstrate a performance enhancement from vitamin D supplementation. However, research examining the aging population (>65 years of age) suggests benefits from vitamin D supplementation. Multiple performance studies in older adults have related low vitamin D levels to decreased reaction time, poor balance, and an increased risk of falling [3]. Furthermore, vitamin D supplementation (800 IU/ day) in older adults showed improvements in strength, and walking distance, and a decrease in general discomfort [3]. These favorable results in older adults support the need for further research on athletic performance and vitamin D. 

The current research available to support vitamin D’s influence on performance is quite limited. An (*n* = 39), unpublished thesis examined 25(OH)D and maximal oxygen uptake (VO_2_max) to determine vitamin D’s effect on aerobic fitness in physically active college males [41]. Higher 25(OH)D levels were associated with an increased VO2max, compared to those with lower vitamin D levels (*p* < 0.01) [41]. These findings suggest that a favorable vitamin D status may improve aerobic performance.

Close *et al.* (2013) examined, young, United Kingdom (UK, 53°N) based athletes (*n =* 30), and examined the effects that vitamin D supplementation (20–40,000 IU/week for 12 weeks) had on muscle performance (1-RM bench press, leg press and vertical jump height) [39]. Subjects were assigned to a placebo, 20,000 IU/week or 40,000 IU/week of vitamin D for 12 weeks. Muscle performance and 25(OH)D was measured at six and 12 weeks, revealing that six weeks of supplementation was enough to correct vitamin D deficiency, however, it was not enough to obtain optimal vitamin D levels >40 ng/mL [39]. Contrary to the findings in the elderly population, no significant improvements in muscle performance were observed after 6 or 12 weeks of vitamin D supplementation, although serum 25(OH)D levels significantly increased over this time, from an average of 20.43 ng/mL to 31.65–39.26 ng/mL [39]. In this study, lower baseline concentrations appeared to respond greater to supplementation, therefore, future studies may find more substantial results by dividing subjects into groups based on their baseline levels. 

Although final 25(OH)D concentrations obtained by the athletes were no longer considered deficient (>20 ng/mL), researchers hypothesized that higher total serum levels may be necessary to document enhanced muscle performance in young athletes [8,39]. According to Close *et al.* (2013), higher 25(OH)D levels may be necessary to induce a physiological response within skeletal muscle [39]. To explain the lack of response, the author suggested that skeletal muscle may require higher serum concentrations for a response, compared to other tissues [39]. The significant response shown in elderly subjects, however, may be explained by sarcopenia. If the elderly were actively losing muscle mass, they may have a more sensitive response to vitamin D supplementation in the skeletal muscle [39]. The authors suggested that more convincing results may be observed by giving supplemental doses of vitamin D to increase serum 25(OH)D above 40 ng/mL. 

A larger (*n* = 61 athletes, *n* = 31 healthy control subjects) UK-based vitamin D supplementation trial resulted in higher mean 25(OH)D levels, as a result of 5000 IU/day of vitamin D3 for eight weeks and found promising muscle performance results [40]. This supplementation regime significantly increased mean 25(OH)D levels from (mean ± SD) 11.62 ± 10.02 ng/mL to 41.27 ± 10.02 ng/mL, whereas a placebo group showed no significant changes. The supplementation group also displayed significant improvements (*p* = 0.008) in 10-meter sprint times and vertical jump (with no improvements in 1-RM bench and squat tests) compared to the placebo group [40]. One athlete’s 25(OH)D levels increased from 22.40 ng/mL to 55.69 ng/mL and showed improvements in all performance areas, this is only one athlete however. These findings support the aforementioned hypothesis that higher serum 25(OH)D levels (>40 ng/mL) may generate more convincing performance improvements [40]. Findings also suggest that a daily dose of vitamin D (5000 IU/day) may be superior in raising 25(OH)D levels when compared to a weekly dose (40,000 IU/week) [39,40]. Based off of these two preliminary studies and guidelines from leading experts, 25(OH)D levels above 40 ng/mL are likely necessary to significantly improve anaerobic athletic performance. There are no studies available that have examined the effect of vitamin D on aerobic or endurance athletic performance. 

To maintain 25(OH)D levels of 40 ng/mL, vitamin D supplementation, especially during the winter months, is warranted [20,28,39,40]. The 25(OH)D goal of 40 ng/mL is recommended for athletes because at this level, vitamin D begins to be stored in the muscle and fat for future use. Furthermore, at levels below 32 ng/mL, vitamin D is not likely to be readily available for the advanced processes involved in the autocrine pathways, which is the pathway that is most likely to influence performance [20,25]. This level is also supported by the two comparable Close *et al.* studies, where the study achieving 25(OH)D levels greater than 40 ng/mL showed significant effects on performance [39,40].

Besides the two UK based performance trials [39,40], recent research on vitamin D and athletes has focused on the prevalence of vitamin D insufficiency among athletes, not the effects on performance. Although performance trials are limited, various other studies have resulted in alternative findings to support vitamin D’s positive impact on performance. Willis *et al.* (2012) revealed that decreased vitamin D was associated with an increased marker for inflammation in endurance athletes [2]. These results call for future investigation to determine whether decreased vitamin D may increase the risk for inflammatory-related injuries [2]. Razavi *et al.* (2011) found that vitamin D and aerobic exercise improved exercise tolerance in asthmatic patients (compared to a control, only aerobic exercise or only vitamin D supplementation groups), suggesting that vitamin D and aerobic exercise together, may provide anti-inflammatory effects within the lungs [42].

As previously mentioned, the body requires an estimated 3000–5000 IU/day of vitamin D and the high levels of physical activity in athletes may result in increased physiological demands for vitamin D [12]. Since vitamin D is actively used in many metabolic pathways, it is possible that the athlete may require increased intake of vitamin D to assure adequate availability and storage for optimal performance [32]. This hypothesis may explain the lack of response observed from Close *et al.*, when 25(OH)D levels above 40 ng/mL were not achieved and may also support increased vitamin D intake recommendations for athletes [40]. At this point, the appropriate vitamin D supplementation regime for athletes appears to depend on current 25(OH)D levels, season and sun exposure, with the goal of >40 ng/mL in mind. Considering these factors, many athletes, especially indoor athletes and those who are insufficient, will require up to 5000 IU of vitamin D/day for eight weeks, to reach 40 ng/mL, then 1000–2000 IU/day for maintenance.

Although the results of performance trials are not yet convincing enough to support vitamin D as a direct performance enhancer, obtaining optimal 25(OH)D levels can reduce the risk of debilitating stress fracture among athletes, which may indirectly influence performance through prevention of injury [8,9]. In addition, because of its active role in muscle, resolution of vitamin D insufficiency has the potential to impact performance [11,14]. 

## 7. Conclusion

Vitamin D is established as a major factor in preventing stress factors and optimizing bone health, both of which are of great importance to the athlete [8,9]. Rates of vitamin D insufficiency in athletes vary among studies, but most researchers agree that athletes should be evaluated regarding vitamin D status and given intake recommendations to maintain optimal 25(OH)D levels >40 ng/mL. Not only does vitamin D assist in growth and maintenance of the bone, but it also aids in regulation of electrolyte metabolism, protein synthesis, gene expression, and immune function [10,28]. These vital functions are essential for all individuals, especially the elite and recreational athlete. Therefore, regardless of the limited literature available in support of a positive effect from vitamin D on performance, obtaining optimal 25(OH)D levels should be a goal for all athletes.

The data are not conclusive to support vitamin D supplementation as a direct performance enhancer, however, research supports the role of vitamin D in the prevention of chronic and acute diseases, such as: cancer, cardiovascular disease, type 2 diabetes, autoimmune diseases and infectious diseases [18]. Athlete or not, optimal vitamin D status is essential to countless fundamental body functions, making it important for all individuals to obtain appropriate levels. Further research is warranted to appropriately define supplementation regimes for specific populations (elderly, athletes, those who are deficient, altering levels for the seasons), establish definite serum 25(OH)D goals, and investigate the effect of vitamin D on physical performance, especially endurance training.

While there is still limited evidence to support vitamin D as a performance enhancer, sports physicians should consider the importance of optimal vitamin D status to prevent stress fractures and muscle injury. Further research is warranted to determine the magnitude of effect from vitamin D on muscle strength and performance. Based off of the prevalence data, high-risk athletes, such as indoor athletes and those who avoid peak daylight hours, should have 25(OH)D levels assessed annually.
